# Hepatoprotective Effect of Nicotinamide Mononucleotide on High‐Fat Diet‐Induced Non‐Alcoholic Fatty Liver Disease in Mice

**DOI:** 10.1002/fsn3.72465

**Published:** 2026-10-28

**Authors:** Yiwen Zhang, Yafei Wu, Yanhong Wei, Xuan Liu, Xiaocan Lei, Hua Huang, Meixiang Li

**Affiliations:** ^1^ Institute of Clinical Anatomy and Reproductive Medicine, Department of Histology and Embryology, Hengyang Medical School University of South China Hengyang Hunan China; ^2^ Youjiang District Ethnic Medicine College Baise City Guangxi Zhuang Autonomous Region China; ^3^ Reproductive Hospital of Guangxi Zhuang Autonomous Region Nanning China

**Keywords:** HNF4α, nicotinamide mononucleotide, non‐alcoholic fatty liver disease, PPAR‐γ, sex hormone‐binding globulin

## Abstract

Non‐alcoholic fatty liver disease (NAFLD) is a significant global public health issue characterized by hepatic lipid accumulation. Nicotinamide adenine dinucleotide (NAD+) is a crucial cofactor in cellular metabolism across mammalian organs. Nicotinamide mononucleotide (NMN), an important intermediate in NAD+ salvage synthesis, may influence high‐fat diet (HFD)‐induced hepatic steatosis. An HFD‐induced NAFLD mouse model was established in C57BL/6 mice, followed by NMN administration for 8 weeks. HepG2 cells were treated with palmitic acid and co‐incubated with NMN to establish an in vitro lipid accumulation model. Network pharmacology analysis and transcriptomic profiling were performed to identify potential molecular targets and pathways involved in the protective effects of NMN against NAFLD. Lipid accumulation was evaluated by H&E staining and Oil Red O staining. Serum lipid metabolism parameters were measured using assay kits. Quantitative RT‐PCR and Western blotting were used to examine the expression of pathway‐related genes and proteins to investigate the potential mechanism by which NMN alleviates hepatic steatosis. Compared with the HFD model group, NMN intervention significantly reduced body weight and alleviated hepatic steatosis. It also lowered levels of low‐density lipoprotein (LDL), triglycerides, total cholesterol, and fasting blood glucose, while increasing high‐density lipoprotein (HDL) levels. Liver PPAR‐γ levels decreased, while HNF4α and SHBG levels increased. These findings suggest that the protective effects of NMN on HFD‐induced hepatic steatosis are associated with decreased hepatic PPAR‐γ expression and increased HNF4α and SHBG expression. NMN showed protective effects against HFD‐induced NAFLD, which were associated with improvements in hepatic lipid metabolism. By integrating network pharmacology, transcriptomic analysis, and experimental validation, this study provides initial evidence regarding molecular changes associated with the effects of NMN in NAFLD.

## Graphical abstract

Nicotinamide mononucleotide (NMN) supplementation alleviated hepatic steatosis in mice with high‐fat diet‐induced non‐alcoholic fatty liver disease, improved serum lipid and fasting glucose profiles, and was associated with decreased hepatic PPAR‐γ expression and increased HNF4α and sex hormone‐binding globulin expression.

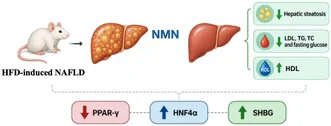

**Abbreviations**
CCK‐8: cell counting kit‐8
CTD: comparative toxicogenomics database
DAB: 3,3′‐diaminobenzidine
DEGs: differentially expressed genes
GAPDH: glyceraldehyde‐3‐phosphate dehydrogenase
GO: gene ontology
H&E: hematoxylin and eosin
HDL‐C: high‐density lipoprotein cholesterol
HFD: high‐fat diet
HNF4α: hepatocyte nuclear factor 4 alpha
HRP: horseradish peroxidase
IHC: immunohistochemistry
KEGG: Kyoto Encyclopedia of Genes and Genomes
LDL‐C: low‐density lipoprotein cholesterol
NAD^+^
: nicotinamide adenine dinucleotide
NAFLD: non‐alcoholic fatty liver disease
ND: normal diet
NMN: nicotinamide mononucleotide
OGTT: oral glucose tolerance test
ORO: Oil Red O
PA: palmitic acid
PBST: phosphate‐buffered saline containing Tween 20
PPAR‐γ: peroxisome proliferator‐activated receptor gamma
PPI: protein–protein interaction
PVDF: polyvinylidene fluoride
qRT‐PCR: quantitative real‐time polymerase chain reaction
RIPA: radioimmunoprecipitation assay
SDS‐PAGE: sodium dodecyl sulfate‐polyacrylamide gel electrophoresis
SHBG: sex hormone‐binding globulin
TC: total cholesterol
TG: triglyceride
WB: Western blotting

## 1 Introduction

NAFLD encompasses a spectrum of liver abnormalities, ranging from simple steatosis to nonalcoholic steatohepatitis (Younossi et al. 2016; Byrne and Targher 2015). NAFLD is characterized by excessive triglyceride accumulation in hepatocytes, while NAFLD also involves inflammation and fibrosis (Friedman et al. 2018; Tilg and Moschen 2010). NAFLD has become one of the most prevalent chronic liver diseases worldwide, affecting approximately 25% of adults, reflecting the increasing prevalence of diabetes and metabolic syndrome (Rinella 2015; Williams et al. 2011).

The course of NAFLD is variable, with NAFLD potentially progressing to cirrhosis and hepatocellular carcinoma, making it a major cause of severe liver disease and a significant public health concern (Paul Angulo 2002). The pathogenesis of NAFLD is complex, involving the interplay of multiple factors including insulin resistance, oxidative stress, lipotoxicity, and chronic inflammation (Peverill et al. 2014; Meli et al. 2014). Despite extensive research into the pathophysiology of NAFLD, there are currently no approved specific treatments (Musso et al. 2009). The efficacy of previously used therapies, such as lipid‐lowering drugs, insulin sensitizers, anti‐hyperglycemic agents, and antioxidants, remains uncertain (Ratziu et al. 2015). This situation underscores the urgent need to explore new therapeutic strategies.

In recent years, NMN, as an important intracellular precursor of NAD^+^, has garnered significant attention from the research community. NAD^+^ is not only a key co‐factor in cellular energy metabolism but also plays a crucial role in regulating oxidative stress responses, DNA repair, and inflammatory reactions (Canto and Auwerx 2012a; Gomes et al. 2013). Studies have shown that NMN, by boosting NAD^+^ levels, possesses potential protective effects against various metabolic diseases such as obesity, diabetes, and aging (Zhang et al. 2016; Yoshino et al. 2011). Specifically, in animal models, supplementation with NMN has shown significant improvements in insulin sensitivity, reduction of oxidative stress, and lowering inflammation levels (Yoshino et al. 2011; Katsyuba et al. 2018). These findings suggest that NMN may exert beneficial effects on NAFLD through multiple pathways, including energy metabolism, oxidative stress, and inflammation regulation. However, the specific role and underlying mechanisms of NMN in NAFLD remain incompletely understood and warrant further in‐depth investigation.

NMN was selected in this study rather than direct NAD^+^ supplementation because NMN is a key intermediate in the NAD^+^ salvage pathway and has been shown to be orally bioavailable and rapidly utilized for NAD^+^ biosynthesis in mammalian tissues (Mills et al. 2016). In hepatic lipid metabolism, PPAR‐γ is involved in lipid uptake and lipid storage, whereas HNF4α is a liver‐enriched transcription factor essential for hepatocyte identity and hepatic metabolic homeostasis (Zaiou 2023). HNF4α also regulates hepatic SHBG expression. SHBG is mainly synthesized in the liver and functions as a carrier of sex steroids, while reduced SHBG levels have been associated with obesity, insulin resistance, Type 2 diabetes, and NAFLD (Xiao et al. 2024; Saez‐Lopez et al. 2017). These findings suggest that the PPAR‐γ/HNF4α/SHBG pathway may be relevant to HFD‐induced hepatic steatosis and the protective effects of NMN.

Accordingly, this study aims to explore the hepatoprotective effects of NMN on HFD‐induced NAFLD in mice and its potential mechanisms. We will first establish a high‐fat‐fed NAFLD mouse model to systematically assess the impact of NMN intervention on mouse mass, blood glucose and lipid levels, and the extent of hepatic steatosis. Building on this, to elucidate the molecular mechanisms by which NMN ameliorates NAFLD, we further employed transcriptomics to deeply analyze the regulatory effects of NMN on hepatic lipid metabolism, oxidative stress, and inflammation signaling pathways. Accordingly, this study aimed to evaluate the hepatoprotective effects of NMN on HFD‐induced NAFLD in mice and to explore the molecular changes associated with NMN treatment. Transcriptomic analysis was performed to characterize changes in pathways related to hepatic lipid metabolism, oxidative stress, and inflammation. We also examined whether NMN treatment was accompanied by changes in hepatic PPAR‐γ, HNF4α, and SHBG expression. This integrated approach was intended to provide insights into the molecular associations underlying the effects of NMN in NAFLD.

## 2 Materials and Methods

### 2.1 Experimental Animals

All experimental procedures were approved by the Animal Ethics Committee of the University of South China (License number: USC 2020031602). Thirty‐six male C57BL/6J mice (4 weeks old, 20 ± 2 g) were obtained from the Experimental Animal Center of the University of South China (Hengyang, China; License number: USC2020031602). The mice were kept in a temperature‐controlled environment (24°C ± 2°C) with a regular dark–light cycle (12 h:12 h) and free access to food and water.

After a week of acclimatization, the mice were randomly divided into three groups (*n* = 12): control group (Ctrl), NAFLD group, and NAFLD+NMN group. Mice in the control group were fed a normal diet (ND), whereas mice in the NAFLD and NAFLD+NMN groups were fed a high‐fat diet (HFD; 60% kcal from fat, composed of 25.8% casein, 0.4% L‐cysteine, 16.1% maltodextrin, 8.9% sucrose, 6.4% cellulose, 3.2% soybean oil, 32% lard, 1.3% mineral mix, 1.6% dicalcium phosphate, 0.7% calcium carbonate, 2.1% potassium citrate H_2_O, 1.3% vitamin mix V10001, and 0.2% choline bitartrate; Product #D12492, Beijing Keao Xieli Feed Co. Ltd., China) for 12 weeks to establish an HFD‐induced NAFLD mouse model, as previously described in C57BL/6 mouse studies of diet‐induced NAFLD (Recena Aydos et al. 2019; Ji et al. 2019).

After the initial 12‐week modeling period, mice in the NAFLD+NMN group continued to receive HFD along with NMN treatment (500 mg/kg/day, dissolved in saline (Ma et al. 2022; Yasuda et al. 2021)) administered by gavage for an additional 8 weeks, while mice in the NAFLD group continued receiving only the HFD for 8 weeks (Liu et al. 2025). The dose of NMN (500 mg/kg/day by oral gavage) was selected based on previous rodent studies using comparable NMN doses in HFD‐induced obesity or metabolic disease models (Liu et al. 2025). Preclinical oral toxicity studies have also suggested that NMN is generally well tolerated at this dose range (Turner et al. 2021; You et al. 2020).

At the end of the 12‐week modeling period, the body weight of mice in the HFD‐fed groups exceeded the average body weight of the control group by approximately 10%–20%, which was used as the primary phenotypic indicator before NMN intervention. All mice were anesthetized by intraperitoneal injection of 20% urethane (0.6 mL/100 g) before euthanasia. Body weights were measured at the end of modeling. Blood samples were collected after anesthesia, and liver tissues were harvested. A portion of liver tissue from mice was fixed overnight in 4% paraformaldehyde and embedded in paraffin, while the remaining liver tissue was stored at −80°C for subsequent molecular analysis.

At the experimental endpoint, H&E staining revealed hepatocellular steatosis, and Oil Red O staining showed increased neutral lipid accumulation in hepatocytes. Compared with the control group, HFD‐fed mice showed elevated serum TG and TC levels, with TG increasing from 0.255 ± 0.039 to 0.443 ± 0.100 mmol/L and TC increasing from 3.312 ± 0.214 to 7.975 ± 1.696 mmol/L, corresponding to increases of approximately 73.7% and 140.7%, respectively. Together, these histological and metabolic alterations supported the successful establishment of the HFD‐induced NAFLD mouse model (Lau et al. 2017; Hansen et al. 2017; Farrell et al. 2019).

### 2.2 Glucose Tolerance Test

At the end of the treatment, mice were fasted overnight and subjected to an oral glucose tolerance test (OGTT). Glucose (2 g/kg body weight) was administered by oral gavage, and blood glucose levels were measured at 0, 30, 60, and 120 min using a glucometer (Accu‐Chek, Roche Diagnostics, Basel, Switzerland) (Andrikopoulos et al. 2008).

### 2.3 Determination of Serum Lipid Metabolism Levels

Serum lipid metabolism levels were measured using commercial assay kits according to the manufacturer's instructions. Blood was collected from the abdominal aorta of mice at the time of euthanasia, kept at room temperature (23°C ± 2°C) for 30 min, and then centrifuged at 2500 rpm for 15 min at 4°C to obtain serum. The concentrations of TC, TG, LDL‐C, and HDL‐C were determined using enzymatic colorimetric assay kits. Briefly, enzymatic reactions generated chromogenic products, and absorbance was measured at 510 nm for TC and TG and at 546 nm for LDL‐C and HDL‐C. The concentrations of TC, TG, LDL‐C, and HDL‐C were calculated according to the standard curves and the manufacturer's protocols (Miller et al. 2010).

### 2.4 Histological Analysis of the Liver

Liver tissues were fixed overnight in 4% paraformaldehyde. Part of the liver samples was embedded in paraffin and sectioned into 4 μm thick slices for hematoxylin and eosin (H&E) staining. H&E staining was performed to evaluate the general histological morphology of the liver and the degree of hepatocellular steatosis, as described in established histological assessment systems for NAFLD and rodent NAFLD (Kleiner et al. 2005).

For ORO staining, another portion of liver tissues was fixed in 4% paraformaldehyde and then sequentially dehydrated in 10%, 20%, and 30% sucrose solutions. The samples were embedded in optimal cutting temperature compound and sectioned into 6–7‐μm‐thick frozen sections. The frozen sections were stained with ORO to visualize neutral lipid accumulation and lipid droplets (Mehlem et al. 2013). Finally, the sections were counterstained with hematoxylin, washed with distilled water, and mounted with glycerol gelatin. Hepatic lipid droplets were observed under a light microscope.

### 2.5 RNA Isolation and Quantitative Real‐Time PCR (qRT‐PCR)

Total RNA was extracted from liver tissues using TRIzol reagent and reverse‐transcribed to cDNA using the TransScript One‐Step gDNA Removal and cDNA Synthesis SuperMix Kit, according to the manufacturer's protocol. For each group, three independent biological samples were analyzed, and each biological sample was tested in technical triplicate. Real‐time PCR analysis was performed using ChamQ Universal SYBR qPCR Master Mix and the QuantStudio 7 Flex Real‐Time PCR System (Thermo Fisher, Waltham, MA, USA). Primers are as listed in the Table 1. Data were normalized to the endogenous GAPDH and quantified using the 2‐ΔΔCt method for fold change calculations. The experiment was repeated at least three times.

**TABLE 1:** Primer sequences used for the quantitative real‐time polymerase chain reaction analysis.

Gene	Primer sequence (5′‐3′)	Accession number
PPAR‐γ	F:GGAAGACCACTCGCATTCCTT R:GTAATCAGCAACCATTGGGTCA	NM_001127330
HNF4a	F:CACGGGCAAACACTACGGT R:TTGACCTTCGAGTGCTGATCC	NM_175914
SHBG	F:GCCCAGGACAAGAGCCTATC R:CCTTAGGGTTGGTATCCCCATAA	NM_001146281

### 2.6 Western Blotting Analysis

Liver tissues were lysed in RIPA buffer (Promega, Madison, WI, USA) and centrifuged at 12,000 rpm for 20 min at 4°C to obtain the supernatant. Protein concentration was measured using the Bradford reagent, followed by the separation of lysates containing equal amounts of protein on a 12% sodium dodecyl sulfate‐polyacrylamide gel electrophoresis (SDS‐PAGE) and transfer to a polyvinylidene fluoride (PVDF) membrane (Millipore, Billerica, MA, USA). After blocking with blocking buffer, the membrane was incubated overnight at 4°C with primary antibodies: anti‐PPAR‐γ (Proteintech, Cat. No. 16643–1‐AP, 1:1000), anti‐HNF4α/HNF4A (Proteintech, Cat. No. 26245–1‐AP, 1:1000), anti‐SHBG (ABclonal, Cat. No. A7450, 1:1000), anti‐GAPDH (Proteintech, Cat. No. 60004–1‐Ig, 1:10000), and anti‐β‐Tubulin (Proteintech, Cat. No. 66240–1‐Ig, 1:10000). The membranes were then incubated with horseradish peroxidase‐conjugated goat anti‐rabbit or goat anti‐mouse IgG secondary antibodies, as appropriate, and dipped into ECL Plus Western Blotting Detection Reagent for chemiluminescence and protein bands detection using the Tanon‐5500 Chemiluminescent Imaging System. Band intensity was quantified using ImageJ software.

### 2.7 Immunohistochemical Analysis

IHC of liver was done on formaldehyde‐fixed, paraffin‐embedded specimens according to the manufacturers' protocols, following the procedure described by Geng et al. (2024). 5 μm‐thick paraffin sections were dewaxed and rehydrated. The antigen was retrieved by microwave treatment and the endogenous peroxidase was inactivated with 5% bovine serum albumin for 45 min. Next, slices were incubated with polyclonal primary rabbit SHBG (1:500 dilution), HNF4α (1:500 dilution), PPAR‐γ (1:500 dilution) overnight at 4°C. After washing with PBST (Phosphate‐buffered saline containing 0.1% Tween 20), the slices were incubated with corresponding secondary antibody at room temperature for 90 min, and then incubated with HRP for 45 min. Antibody bound to the section was visualized in DAB solution, hematoxylin counter‐staining. Normal serum was used as a negative control. Finally, the stained sections were observed and photographed under a light microscope (BX43, Olympus). Immunostaining and histological features were independently evaluated by two experienced authors, who were blinded to the group allocation during assessment.

### 2.8 Identification of Potential NMN Targets

Potential pharmacological targets of NMN were identified through systematic database queries. First, the Comparative Toxicogenomics Database (CTD; https://ctdbase.org/) was searched using “nicotinamide mononucleotide” as the chemical keyword. Parallel searches were conducted in the SwissTargetPrediction database (http://www.swisstargetprediction.ch/) with identical nomenclature. The NMN‐associated targets retrieved from both databases were consolidated, standardized according to their official gene symbols, and deduplicated using Excel 2021 (Microsoft Corporation).

### 2.9 Disease Target Selection

NAFLD‐associated targets were acquired from the GeneCards database (https://www.genecards.org/) by querying “non‐alcoholic fatty liver disease” OR “NAFLD” with a relevance score filter set at ≥ 10 points. To identify candidate therapeutic targets, NMN‐associated targets (*n* = 60) and NAFLD‐related targets (*n* = 2479) were cross‐analyzed using Venny 2.1.0, yielding 38 overlapping targets through Venn diagram intersection.

### 2.10 Protein–Protein Interaction (PPI) Network Construction

The 38 candidate targets were subjected to PPI network analysis via STRING v11.5 (https://string‐db.org/) under *Homo sapiens* species restriction, with interaction confidence scores > 0.4 and disconnected nodes hidden. As MIR34A encodes a non‐protein‐coding microRNA and could not be represented as a protein node in STRING, the remaining 37 protein‐coding targets were included in the PPI network. The resultant network (TSV format) was imported into Cytoscape 3.9.1 for visualization using force‐directed layout algorithms. Topological properties were systematically calculated through integrated NetworkAnalyzer tools, including six centrality metrics: Betweenness Centrality (BC), Closeness Centrality (CC), Degree Centrality (DC), Eigenvector Centrality (EC), Network Centrality (NC), and Local Average Connectivity (LAC). Core targets were defined as nodes ranking in the top 15% across multiple parameters (BC > 0.008, CC > 0.45, DC > 12) using the cytoNCA plugin.

## 3 Result

### 3.1 Network Pharmacology Analysis of NMN in NAFLD

We identified 2479 genes associated with NAFLD from database screening. The intersection of NMN targets and NAFLD‐related genes yielded 38 overlapping targets (Figure 1A). Because MIR34A encodes a non‐protein‐coding microRNA and therefore could not be represented as a protein node in STRING, the remaining 37 protein‐coding targets were included in the PPI analysis. The resulting network comprised 37 nodes and 266 edges (Figure 1B). The top five nodes ranked by degree were PPARG, TNF, IL1B, SIRT1, and PPARGC1A, suggesting their central roles in the PPI network.

**FIGURE 1:**
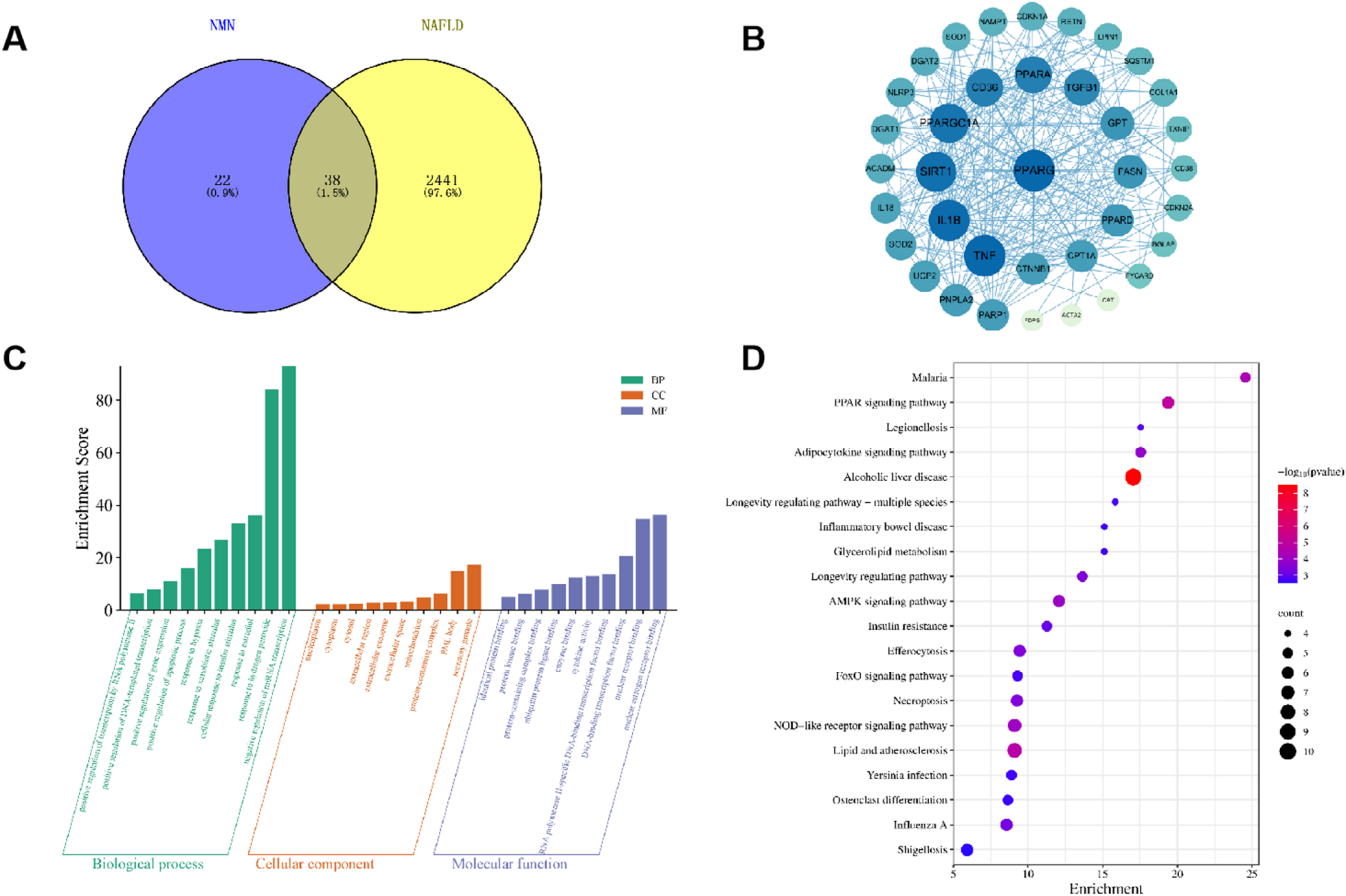
(A) Venn diagram of NMN and NAFLD targets. (B) Key targets (C) Gene Ontology enrichment analysis (visualize the top 10 for each of Biological Process, Cellular Component, and Molecular Function). (D) KEGG pathway map.

Gene Ontology (GO) enrichment analysis using the DAVID database (Figure 1C) revealed 321 enriched terms: 271 in Biological Process (BP), 16 in Cellular Component (CC), and 34 in Molecular Function (MF). Key BP terms included negative regulation of apoptosis, positive regulation of cell proliferation and protein phosphorylation, and positive regulation of endothelial cell proliferation. MF primarily involved enzyme binding, receptor binding, and protease binding. CC mainly included mitochondria, protein complexes, and intracellular membrane‐bound organelles.

KEGG pathway enrichment analysis (Figure 1D) identified 57 signaling pathways. The top 20 pathways, ranked by p‐value, are visualized in a bubble chart. The y‐axis represents different KEGG pathways, bubble size indicates gene count, and the x‐axis shows fold enrichment. Color intensity represents‐lg(P).

### 3.2 NMN Supplementation Reduces HFD‐Induced NAFLD and Glucose Intolerance

To investigate the effects of NMN on NAFLD, we first evaluated the body mass and glucose tolerance of HFD‐fed mice with and without NMN supplementation. Compared to control mice, those fed with HFD for 12 weeks showed a significant increase in body mass. However, NMN supplementation significantly reduced the body mass gain in HFD‐fed mice, indicating an anti‐obesity effect of NMN (Figure 2A–C). At the same time, the OGTT results showed that the mice of NAFLD group had significantly higher blood glucose levels at 30, 60, and 120 min after glucose administration compared to the mice of control group, indicating the development of glucose intolerance. NMN supplementation significantly reduced fasting blood glucose in HFD‐fed mice, though glucose tolerance during the dynamic phase of OGTT remained comparable to untreated NAFLD mice (Figure 2D,E). These results suggest that NMN is a potential therapeutic agent for treating HFD‐induced impairment in food intake and glucose tolerance. As expected, the mice in NAFLD group exhibited a significant increase in abdominal fat mass compared to normal mice. NMN treatment significantly reduced the mass of abdominal fat, Lee index, the diameter and area of adipose tissue in obese mice (Figure 2F–I).

**FIGURE 2:**
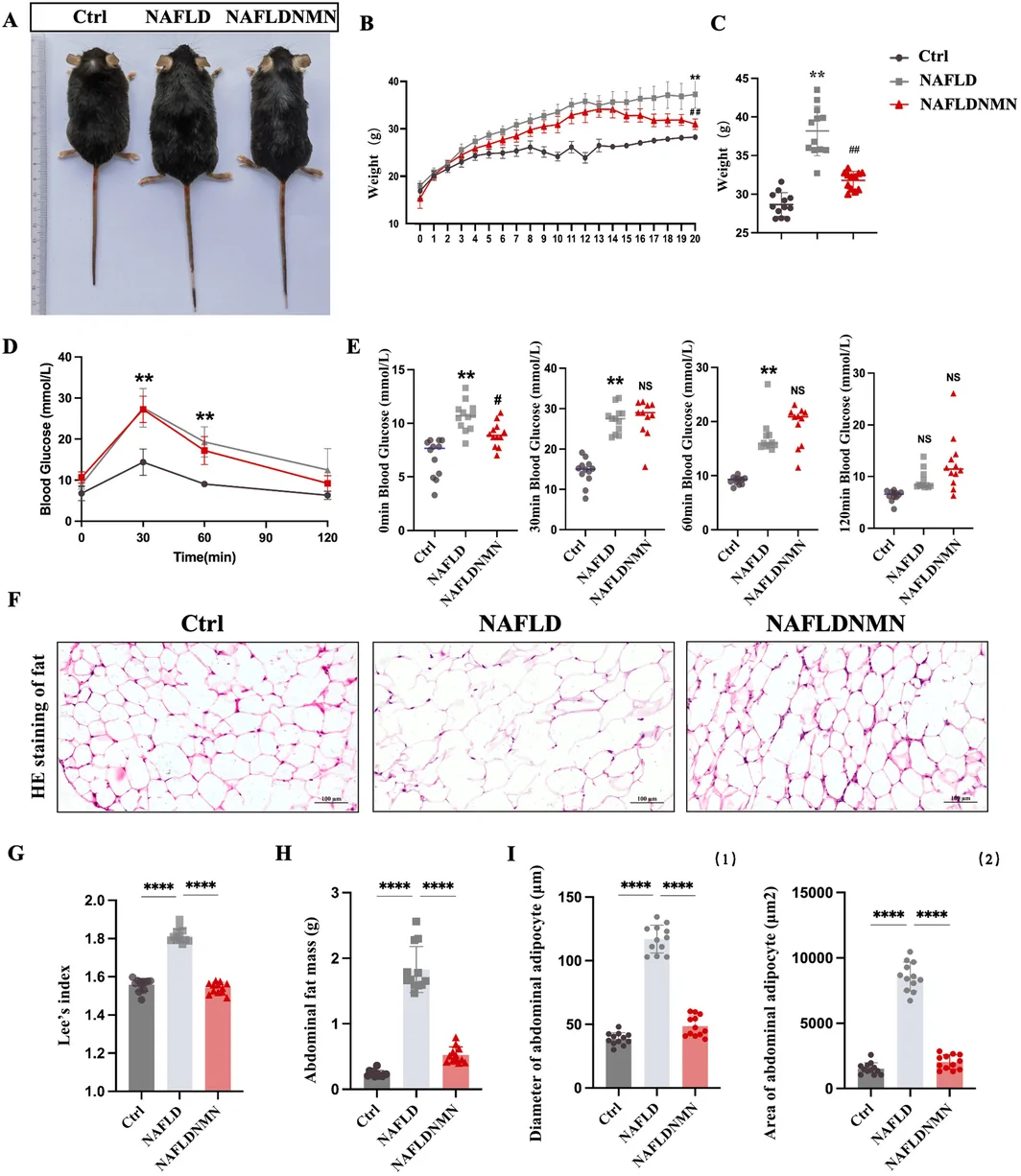
NMN ameliorates the abnormal lipid metabolism in obese mice. (A) Representative appearance of mice at end of the study. (B) The body mass in days on diets and treatment. (*n* = 12). (C) The body mass after treatment (*n* = 12). (D) Oral glucose tolerance test (OGTT) in obese mice. (E) The blood glucose levels at 0, 30, 60, and 120 min during the oral glucose tolerance test (OGTT). (F) The representative light microscopic histology images of abdominal adipose masses stained with hematoxylin and eosin (HE). (G) Relative LEE's index. (H) Abdominal fat mess weight. (I) The diameter (1) and area (2) of abdominal adipocyte in the mice.*p < 0.05, **p < 0.01.

### 3.3 NMN Improves Hepatic Steatosis and Liver Function in Mice

Next, the effects of NMN on serum lipid metabolism were evaluated. As shown in Figure 3, mice in the NAFLD group exhibited significantly higher serum levels of TC, TG, and LDL‐C compared to the control group, but lower HDL‐C levels compared with the control group. Notably, NMN supplementation significantly reduced serum TC, TG, and LDL‐C levels (Figure 3A–D). These results suggested that NMN can improve HFD‐induced dyslipidemia. To determine the impact of NMN on HFD‐induced NAFLD, we performed histological analyses of liver tissues. H&E staining further confirmed the development of hepatic steatosis in HFD‐fed mice (Figure 3E). Liver sections from the control group showed relatively preserved hepatic cord architecture, regularly arranged hepatocytes, and few obvious lipid vacuoles. In contrast, liver tissues from the NAFLD group exhibited marked hepatocellular steatosis, as evidenced by numerous clear vacuole‐like structures of varying sizes within hepatocytes, hepatocellular swelling, and displacement of nuclei, indicating substantial lipid accumulation. After NMN supplementation, the NAFLD+NMN group showed an improved hepatic histological appearance compared with the NAFLD group, with fewer lipid vacuoles, reduced hepatocellular swelling, and relatively more preserved hepatocyte morphology. Consistently, Oil Red O staining showed increased neutral lipid deposition in the livers of NAFLD mice, whereas NMN treatment visibly reduced hepatic lipid droplet accumulation (Figure 3F). These histological findings suggest that NMN supplementation alleviated HFD‐induced hepatic steatosis and NAFLD‐associated liver injury.

**FIGURE 3:**
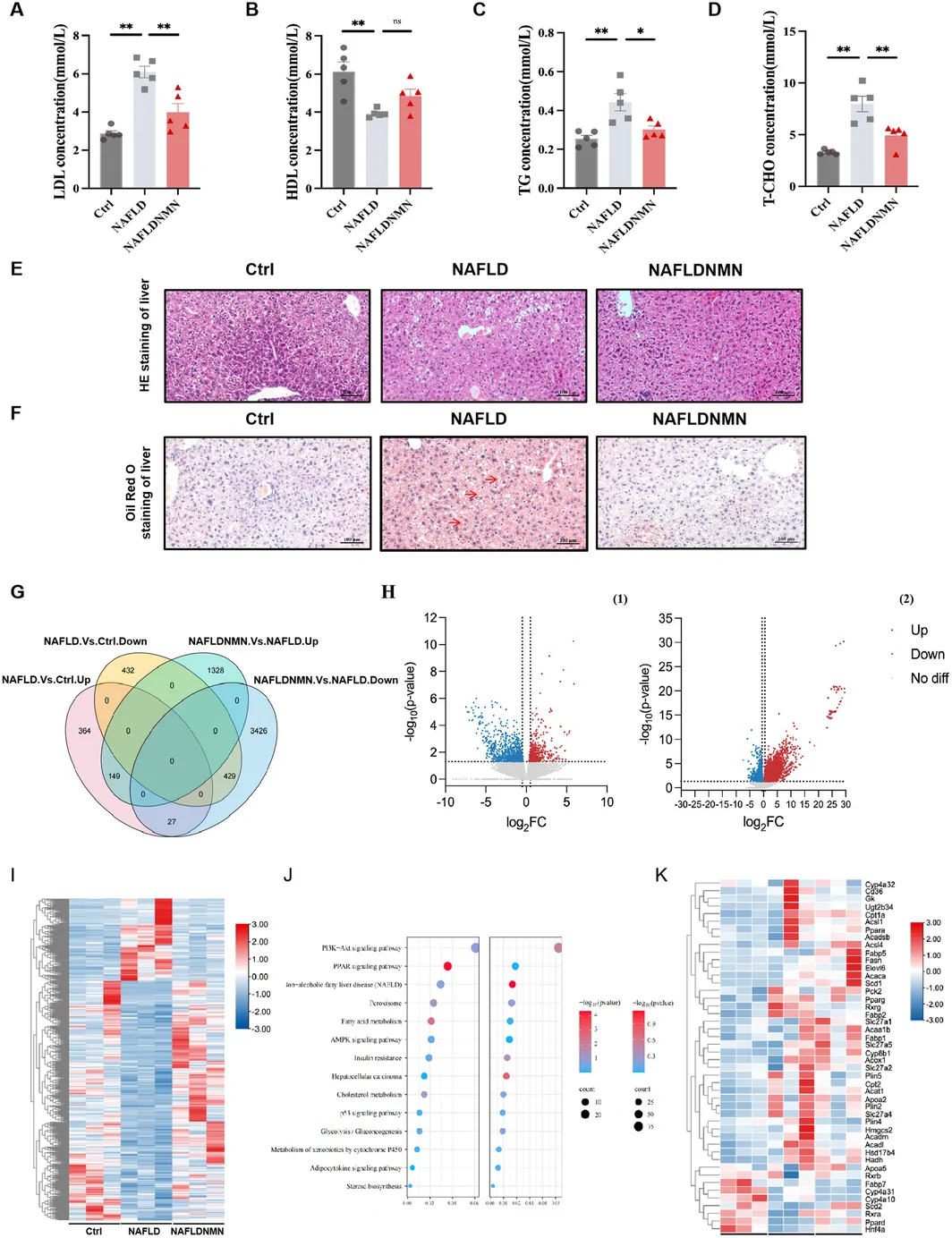
NMN improves hepatic steatosis and abnormal lipid metabolism in mice. (A) Levels of LDL. (B) Levels of HDL. (C) Levels of TG. (D) Levels of T‐CHO. (E) HE staining of liver. (F) Hepatic lipid droplets were visualized by oil red O staining. The red arrow indicates fat droplet. (G) The venn diagram of DEGs. (H) Volcano plots of liver DEGs in NAFLD vs. Ctrl groups (1) and NAFLD+NMN vs. NAFLD groups (2) (*n* = 5). (I) Cluster analysis of differentially expressed genes (DEGs) which are consistently effective treatment of NMN enriched in mice's liver (*n* = 3). (J) KEGG Functional enrichment analysis of DEGs. (K) Cluster analysis of DEGs enriched in lipid metabolism and steroid hormone synthesis pathway. *p < 0.05, **p < 0.01.

### 3.4 Transcriptomic Analysis of Mouse Liver

To explore the molecular mechanisms behind NMN's liver protective effects, RNA sequencing analysis were performed on liver tissues of the control group, NAFLD group and NAFLDNMN groups (Figure 3G). Differential expression analysis identified 1371 DEGs between the NAFLD and control groups, including 540 upregulated and 831 downregulated genes, whereas 5359 DEGs were detected between the NAFLD+NMN and NAFLD groups, including 3882 upregulated and 1477 downregulated genes (Figure 3H). To further characterize the transcriptional alterations associated with HFD‐induced hepatic steatosis and their modulation by NMN, we focused on 578 genes that were differentially expressed among the three groups. Hierarchical clustering of these genes showed that the NAFLD group exhibited a distinct dysregulated transcriptomic profile compared with the control group, whereas NMN supplementation partially shifted the expression pattern toward that of the control group (Figure 3G,I). KEGG pathway enrichment analysis was then performed to interpret the biological functions of these DEGs (Figure 3J; Kanehisa et al. 2023). The enriched pathways were mainly associated with energy metabolism, inflammatory signaling, lipid metabolism, and steroid biosynthesis, which are closely related to hepatic metabolic homeostasis and NAFLD pathogenesis (Stefan et al. 2025; Zhang and Chen 2024). DEGs were enriched in pathways related to energy metabolism, inflammatory signaling, lipid metabolism, and steroid biosynthesis.

The cluster tree of DEGs significantly restored in the NAFLDNMN group showed a down‐regulation in the expression of PPAR‐γ, which mediates the lysosomal degradation of LDL to cholesterol, in response to NMN treatment. Proteins related to lipid metabolism, such as LDLR and steroid synthesis‐related HNF4a, were upregulated in response to HFD but mitigated by NMN intervention (Figure 3K). These results are consistent with the histopathological changes observed in liver tissues due to HFD.

### 3.5 NMN Treatment Is Associated With Changes in Hepatic PPAR‐γ, HNF4α, and SHBG Expression

Based on our RNA‐seq findings, we further explored NMN's effects on the PPAR‐γ/HNF4α/SHBG pathway, which is closely related to hepatic lipid metabolism and glucose homeostasis. As shown in Figure 4C, qRT‐PCR analysis confirmed that PPAR‐γ mRNA expression was significantly upregulated in the livers of NAFLD mice compared to controls, while HNF4α and SHBG mRNA levels were downregulated. Notably, NMN supplementation significantly suppressed PPAR‐γ expression and enhanced HNF4α and SHBG expression in the livers of NAFLD mice.

**FIGURE 4:**
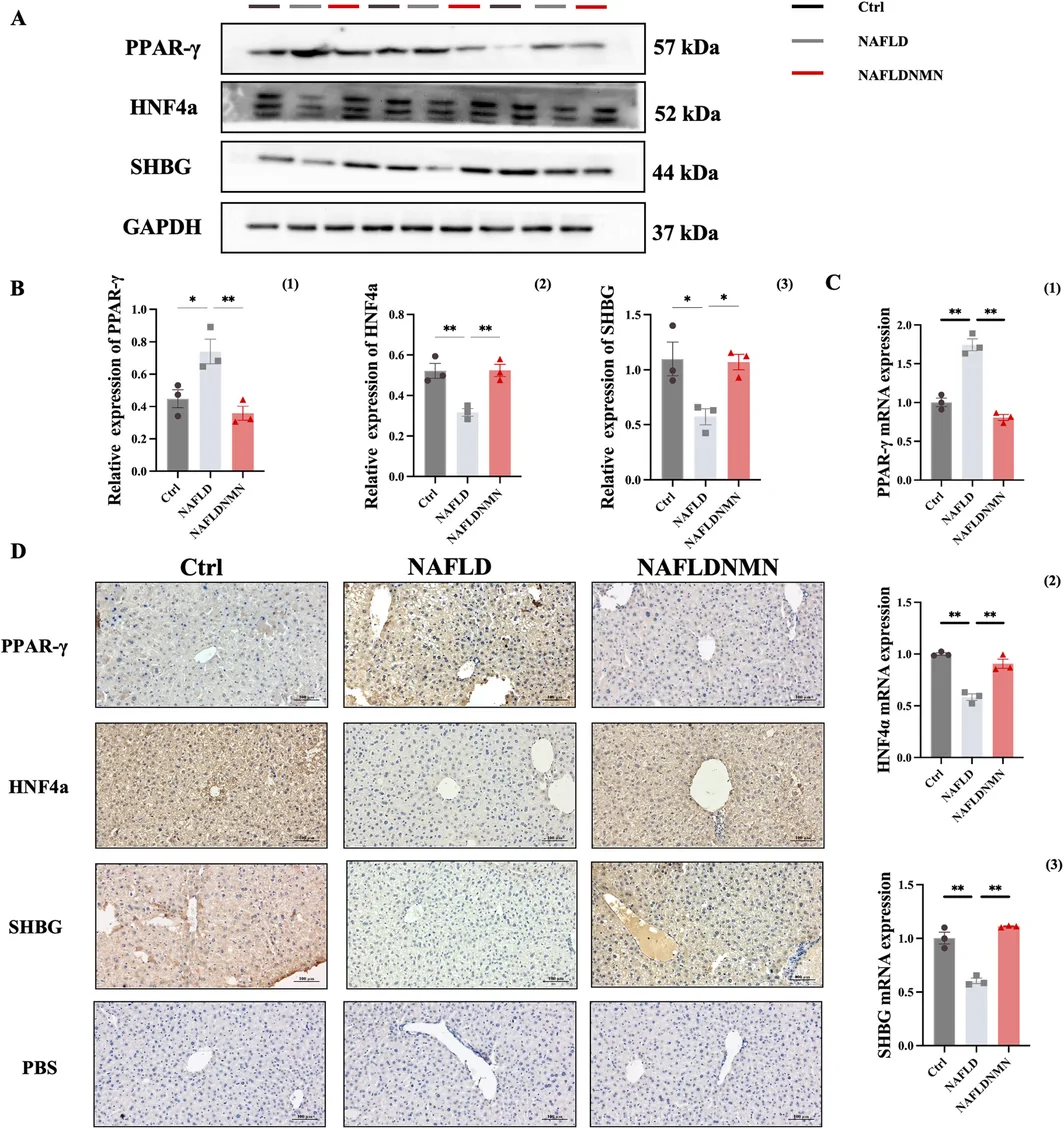
NMN improves lipid metabolism in NAFLD mice. (A) Expression of PPAR‐γ, HNF4a and SHBG in liver detected by WB analysis. (B) Expression of PPAR‐y, HNF4a and SHBG in liver detected by WB analysis. (C) qRT‐PCR validation of PPAR‐γ, HNF4a and SHBG in liver (*n* = 3). (D) Immunohistochemistry analysis of the expression of PPAR‐γ, HNF4a and SHBG in liver. PBST was used as the negative control. *p < 0.05, **p < 0.01.

Consistent with the mRNA results, Western blot analysis revealed that NMN supplementation significantly reduced PPAR‐γ protein levels and increased HNF4α and SHBG protein levels in the livers of NAFLD mice (Figure 4A,B).

Immunohistochemical analysis showed increased PPAR‐γ expression and decreased HNF4α and SHBG expression in liver tissues of NAFLD mice compared with controls. NMN supplementation reduced PPAR‐γ expression while restoring HNF4α and SHBG expression in HFD‐fed mice (Figure 4D). These results indicate that the improvements observed after NMN supplementation were accompanied by decreased hepatic PPAR‐γ expression and increased HNF4α and SHBG expression. These findings support an association between these expression changes and the effects of NMN.

### 3.6 NMN Treatment Is Associated With Changes in PPAR‐γ, HNF4α, and SHBG Expression in PA‐Treated HepG2 Cells

To simulate the high‐fat environment in NAFLD patients, we exposed Human Hepatocellular Carcinoma Cell Line G2 (HepG2 cells) to high concentrations of palmitic acid (PA). This model allowed us to investigate the protective effects of NMN on PA‐induced HepG2 cell damage through the regulation of the PPAR‐γ signaling pathway. We first assessed HepG2 cell viability using the CCK‐8 assay after treatment with various PA concentrations (0, 0.1, 0.2, 0.4, and 0.8 mM) (Figure 5A). Results showed that PA at 0.4 and 0.8 mM reduced cell viability in a dose‐dependent manner compared to the control group. Notably, 0.4 mM PA decreased cell viability to approximately 50%. Based on these findings and previous literature, we selected 0.4 mM PA for subsequent experiments. Next, we evaluated the effect of different NMN concentrations (0, 10, 25, 50, and 100 μM) on HepG2 cell viability (Figure 5B). No significant changes in cell viability were observed across the tested NMN concentrations. We then treated HepG2 cells with 0.4 mM PA and various concentrations of NMN (0, 10, 25, 50, and 100 μM). All tested NMN concentrations improved the viability of PA‐treated HepG2 cells, with 50 μM NMN showing the most significant improvement (Figure 5C). Consequently, we chose 50 μM NMN as the optimal concentration for further experiments.

**FIGURE 5:**
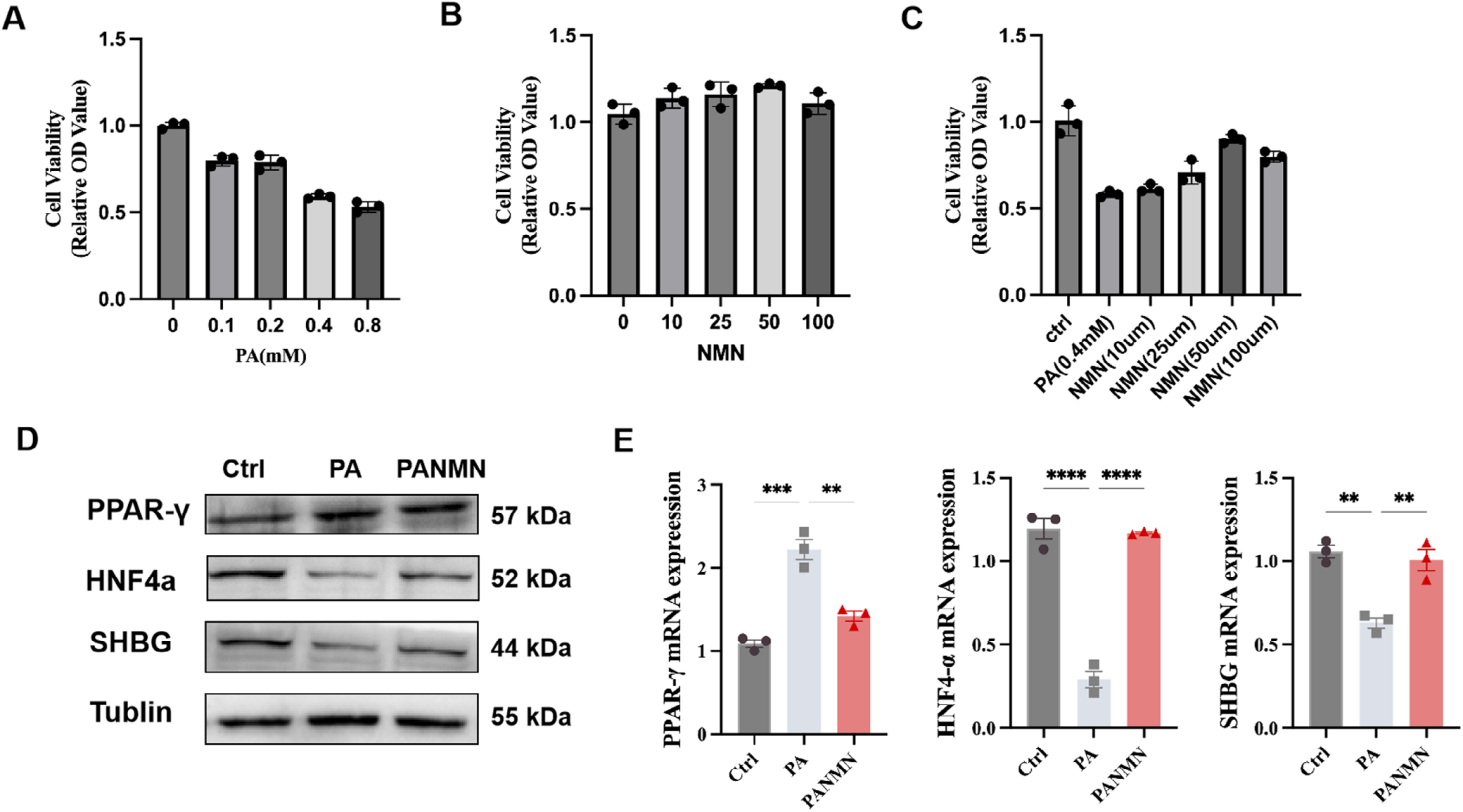
NMN ameliorates NAFLD by inhibiting PPAR‐γ‐mediated lipid metabolism in HepG2 cells. (A) The effect of PA on HepG2 cells proliferation (*n* = 3). (B) The effect of NMN on HepG2 cell proliferation (*n* = 3). (C) The effect of PA and NMN on HepG2 cell proliferation (*n* = 3). (D) Expression of PPAR‐γ, HNF4α and SHBG in HepG2 cell detected by WB analysis. (E) qRT‐PCR validation of PPAR‐γ, HNF4α and SHBG in HepG2 cell (*n* = 3). *p < 0.05, **p < 0.01.

Western blot analysis revealed that the PA + NMN group significantly reduced PPAR‐γ protein levels in PA‐treated HepG2 cells while increasing HNF4α and SHBG protein levels (Figure 5D). As shown in Figure 5E, qRT‐PCR analysis confirmed that compared to the control group, PA treatment significantly upregulated PPAR‐γ mRNA expression in HepG2 cells, while downregulating HNF4α and SHBG mRNA expression. NMN supplementation markedly suppressed PPAR‐γ expression and enhanced HNF4α and SHBG expression in PA‐treated cells (Li et al. 2024). These findings are consistent with our in vivo experimental results.

## 4 Discussion

NAFLD has become the most prevalent chronic liver disease worldwide, with its incidence rising significantly over the past few decades. Recent studies estimate that approximately 25% of adults globally have NAFLD, with this proportion reaching 70%–80% among obese individuals and those with Type 2 diabetes (Younossi et al. 2016; Li et al. 2024). The pathogenesis of NAFLD is complex, involving multiple factors such as insulin resistance, oxidative stress, and lipid metabolism dysregulation (Peverill et al. 2014). In recent years, nicotinamide mononucleotide (NMN), a precursor of NAD^+^, has shown great potential in improving metabolic disorders and delaying aging. However, the role of NMN in NAFLD and its molecular mechanisms remain unclear. In this study, we investigated the protective effects of NMN on HFD‐induced NAFLD and examined whether these effects were associated with changes in hepatic PPAR‐γ, HNF4α, and SHBG expression. By combining network pharmacology analysis with animal and cellular experiments, we seek to provide new insights and experimental evidence for the potential application of NMN in NAFLD prevention and treatment.

In this study, we found NMN supplementation significantly reduced body mass gain and abdominal fat accumulation in HFD‐fed mice, which is consistent with previous findings. These findings suggest that NMN is a potential anti‐obesity agent. HFD‐fed mice exhibited significant dyslipidemia, characterized by elevated serum levels of total cholesterol, triglycerides, and LDL‐C, along with decreased HDL‐C levels. Notably, NMN supplementation significantly reduced serum total cholesterol, triglycerides, and LDL‐C levels while increasing HDL‐C levels in HFD mice, indicating that NMN supplementation can ameliorate HFD‐induced dyslipidemia. Uddin et al. reported that NMN could decrease body mass, white adipose tissue mass, and adipocyte size in HFD‐fed mice by enhancing mitochondrial function and promoting fatty acid oxidation (Uddin et al. 2017). Moreover, a clinical trial by Yoshino et al. demonstrated that NMN positively influenced body composition and fat distribution in healthy overweight or obese women (Yoshino et al. 2021), supporting the potential of NMN as an anti‐obesity supplement. Previous studies have suggested that NMN may improve lipid metabolism by replenishing NAD^+^ levels, influencing NAD^+^‐dependent metabolic regulators such as SIRT1, and modulating energy‐sensing pathways such as AMPK, thereby suppressing lipogenic gene expression and promoting fatty acid oxidation (Canto et al. 2012; Wang et al. 2018; Gariani et al. 2016). In the present study, NMN supplementation reduced hepatic lipid accumulation and improved serum lipid profiles in HFD‐fed mice, which is consistent with a potential role of NAD^+^‐related metabolic regulation. However, hepatic NAD^+^ levels, SIRT1 activity, and AMPK activation were not directly measured in this study; therefore, we cannot conclude that the hepatoprotective effects of NMN were mediated exclusively through NAD^+^ modulation. The OGTT results showed that HFD‐fed mice exhibited elevated blood glucose levels after glucose loading, indicating impaired glucose tolerance. In contrast, NMN supplementation improved glucose tolerance in the NAFLD+NMN group, as evidenced by lower blood glucose levels at the measured time points. Related studies have also shown that NMN can enhance insulin signaling and glucose utilization (Canto and Auwerx 2012b; Yoshino et al. 2011) through increasing NAD^+^ levels, activating SIRT1 and AMPK signaling pathways.

Histological analysis revealed severe hepatic steatosis with numerous lipid droplets in the hepatocytes of NAFLD mice. In contrast, NMN supplementation significantly alleviated hepatic steatosis in HFD mice. Related studies have shown that NMN reduces hepatic lipid accumulation by inhibiting lipogenesis and promoting lipolysis (Gariani et al. 2017; Trammell and Brenner 2013). These histological findings suggest that NMN supplementation can alleviate HFD‐induced hepatic steatosis and NAFLD‐associated liver injury. Additionally, NMN has been shown to exert anti‐inflammatory and antioxidant effects, which can mitigate inflammation and oxidative stress during the progression of NAFLD (Trammell et al. 2016).

In our study, transcriptomic analysis was applied to characterize the transcriptional changes associated with the effects of NMN on HFD‐induced hepatic metabolic dysregulation. RNA sequencing analysis was performed on liver tissues of the mice in the control, NAFLD, and NAFLDNMN group. Differentially expressed genes (DEGs) and conducted functional enrichment analysis were identified. KEGG pathway enrichment analysis revealed that these DEGs were primarily involved in energy metabolism, inflammatory signaling pathways, lipid metabolism, and steroid biosynthesis (Figure 3D). In the NAFLDNMN group, the DEGs in these pathways were significantly restored, suggesting that NMN can ameliorate HFD‐induced liver metabolic dysregulation by regulating these metabolic pathways (Hong et al. 2020).

Our study provides new insights into the molecular changes associated with the protective effects of NMN against NAFLD. HFD feeding was associated with increased PPAR‐γ expression and decreased HNF4α and SHBG expression, whereas NMN supplementation was accompanied by changes in the opposite direction. These coordinated expression changes suggest a potential association between the PPAR‐γ/HNF4α/SHBG pathway and the effects of NMN.

The interplay between PPAR‐γ and HNF4α is crucial in hepatic lipid metabolism. PPAR‐γ promotes fat synthesis and storage, while HNF4α enhances fatty acid oxidation and lipid transport (He et al. 2003; Xiao et al. 2024). Our results showed that the improvement in HFD‐induced hepatic metabolic dysregulation after NMN treatment was accompanied by decreased PPAR‐γ expression and increased HNF4α expression. This balance is essential for maintaining hepatic lipid homeostasis, as evidenced by studies showing exacerbated hepatic steatosis in liver‐specific HNF4α‐deficient mice (Parviz et al. 2003).

Furthermore, our findings highlight the importance of SHBG in this pathway. HNF4α directly activates SHBG transcription (Selva and Hammond 2009; Jänne and Hammond 1998), and some results show that NMN increases SHBG expression, likely through HNF4α upregulation (Selva and Hammond 2009; Yoshino et al. 2011; Ding et al. 2017). This is significant because SHBG has been associated with reduced risk of NAFLD in epidemiological studies (Peter et al. 2010; Flechtner‐Mors et al. 2014). Previous studies have proposed that SHBG may influence hepatic lipid and glucose metabolism by binding to steroid hormones and activating intracellular signaling pathways (Rosner et al. 2010; Simo et al. 2015).

Epidemiological studies have consistently shown a negative correlation between serum SHBG levels and the risk of NAFLD (Peter et al. 2010; Hua et al. 2014). In patients with NAFLD, serum SHBG levels are significantly lower than in normal controls, and SHBG levels are negatively correlated with liver fat content, inflammation, and fibrosis (Saez‐Lopez et al. 2017; Flechtner‐Mors et al. 2014). These findings further support the potential protective role of SHBG in NAFLD development and progression.

These findings extend our understanding of the molecular changes associated with the protective effects of NMN in NAFLD beyond its known role as an NAD+ precursor. The improvements in hepatic lipid accumulation, serum lipid profiles, and glucose homeostasis after NMN treatment were accompanied by coordinated changes in PPAR‐γ, HNF4α, and SHBG expression, supporting a potential association between this pathway and the effects of NMN.

Although our study has provided valuable insights into the protective role of NMN in NAFLD, several limitations should be considered. Firstly, we only tested one dosage of NMN (500 mg/kg/day); dose–response studies are necessary to determine the optimal therapeutic dosage. Secondly, this study mainly examined changes in PPAR‐γ, HNF4α, and SHBG expression, whereas other potential mechanisms, such as inflammation, oxidative stress, and mitochondrial function, were not extensively investigated. Future studies should further explore these additional mechanisms. Finally, this study was conducted solely in animal models; clinical trials are essential to evaluate the efficacy and safety of NMN supplementation in human subjects with NAFLD.

## 5 Conclusion

NMN can ameliorate high‐fat‐induced hepatic steatosis through regulation of the PPAR‐γ/HNF4α/SHBG pathway, modulating serum lipid and blood glucose levels, thereby mitigating the liver damage caused by NAFLD.

## Author Contributions

**Yiwen Zhang:** conceptualization, writing – original draft, validation. **Yafei Wu:** methodology. **Yanhong Wei:** software. **Xuan Liu:** data curation. **Xiaocan Lei:** supervision. **Hua Huang:** supervision, writing – review and editing, funding acquisition. **Meixiang Li:** writing – review and editing, supervision.

## Funding

This study was supported by funding from the Guangxi Natural Science Foundation, China (2026GXNSFAA00640661), the Natural Science Foundation of Hunan Province, China (2026JJ50562), Scientific research project of Hunan Provincial Department of Education (25A0333).

## Conflicts of Interest

The authors declare no conflicts of interest.

## Data Availability

The data supporting the findings of this study are included in this article. Additional raw data are available from the corresponding author upon reasonable request.
